# Implementation of wearable activity trackers in hospital rehabilitation: a feasibility study tailored to local settings

**DOI:** 10.1186/s12913-026-14066-4

**Published:** 2026-01-24

**Authors:** Kimberley Szeto, John Arnold, Ben Singh, Peter Diestel-Feddersen, Dominique Edwards, Shannon Cheary, Matthew Lo Basso, Carol Maher

**Affiliations:** 1 Alliance for Research in Exercise, Nutrition and Activity (ARENA), Adelaide University, GPO Box 2471, Adelaide, SA 5001 Australia; 2 Division Rehabilitation, Aged Care & Palliative Care, Southern Adelaide Local Health Network, Adelaide, South Australia Australia

**Keywords:** Wearable activity tracker, Physical activity, Rehabilitation, Hospital, Inpatient, Outpatient, Implementation, Feasibility, Fitbit

## Abstract

**Background:**

Physical inactivity during and after hospitalisation is associated with poorer outcomes. Wearable activity trackers (WATs) are effective in promoting physical activity in healthcare settings, however, their successful implementation remains a challenge, and evidence for how this can be achieved is so far limited.

**Objective:**

This feasibility study evaluated WAT implementation in inpatient and outpatient rehabilitation by assessing: (1) feasibility metrics including uptake and retention rates; (2) patient and clinician perceptions; (3) challenges and factors contributing to success; and (4) changes in PA.

**Methods:**

A single-arm, feasibility study was conducted in two rehabilitation settings at a major teaching hospital. Patients were provided with a WAT (Fitbit Inspire 3) to wear throughout their admission, which was used to set step goals with clinicians, monitor daily step counts and support efforts and encouragement to meet step goals. Feasibility was assessed based on recruitment, retention, and data completeness rates. Patient and clinician experiences, satisfaction and perceptions were evaluated through surveys. Effect sizes for change in physical activity from baseline to follow-up were assessed for daily step counts, light physical activity, moderate-vigorous physical activity, and sedentary behaviour, were calculated using Cohen’s *d* and matched-pairs rank-biserial correlation (r).

**Results:**

Of 36 invited patients, 26 participated, (mean age 72 years; SD: 13; range 33–93; 72% uptake rate). Data completeness was high (92%), with no devices lost or broken. Most patients perceived WATs helped increase walking (68%) and achieve rehabilitation goals (64%), with 84% willing to use WATs again in rehabilitation. All clinicians (*n* = 6) found WATs easy to use, though perspectives on therapy enhancement varied. Large effect sizes were observed for increased daily steps in both the inpatient (1050 to 1366 steps/day, (r) = 0.595) and outpatient (4749 to 5986 steps/day, (r) = 0.532) rehabilitation settings. The simplicity of the approach and patient and clinician resources were identified as factors contributing to the success of implementation, though the recruitment approach revealed challenges related to inviting individual patients to participate.

**Conclusion:**

WAT implementation was feasible in both rehabilitation settings. Future research should evaluate larger scale implementation and effectiveness of such approaches through service-level enrolment and larger controlled trials, and exploring applications across diverse clinical settings.

**Trial registration:**

This trial was registered with the Australian New Zealand Clinical Trials Registry (ANZCTR) (Trial ID: ACTRN12624000447550; registration date: 11 April 2024).

**Supplementary Information:**

The online version contains supplementary material available at 10.1186/s12913-026-14066-4.

## Introduction

Physical activity (PA) is recognised as an important component of recovery across the healthcare continuum, including during and after a hospital stay, and it is associated with a variety of beneficial clinical and economic outcomes [1–4]. Observational studies show that modest daily step counts (900 steps per day) during a hospitalisation are correlated with better functional recovery trajectories among older adults [5], and just 250–500 daily steps have been associated with shorter lengths of stay, lower risk of 30-day readmission, and discharge to home following cardiac surgery [6]. Patients who perform more PA are more likely to make a full recovery [7], have a lower mortality risk [8], and are less likely to be readmitted [9]. Risks of inactivity extend beyond discharge from inpatient care, with lower PA levels being associated with increased risk of functional decline [10], which affects approximately 70% of older adults following hospitalization for medical illness [11]. These relationships have been identified largely from heterogeneous cohorts (typically mixed medical–surgical, acute and stroke populations), and mainly comprise older adults [3–12], but provide broad insight of the importance of PA during and following a hospital admission, including rehabilitation. Despite the benefits of PA, most patients are extremely inactive during a hospital stay [13, 14], and continue to have low PA levels following discharge from hospital wards, where most do not meet the recommended guideline of 150 min of PA per week [15]. This highlights an urgent need for innovative and effective strategies that address patient PA during and after a hospitalisation, which are feasible for real-world implementation.

One way that patient PA can be improved during and after hospitalisations is by using wearable activity trackers (WATs). Wearable activity trackers are body-worn devices (often on the wrist) that measure the wearer’s movement and allows them to track PA in the form of metrics like step counts or minutes of PA at different intensities. Tracking these metrics can support behaviour change techniques, like goal setting and feedback, and provide dual benefits of giving clinicians insight into patient activity levels, as well as promoting patient PA during care delivery. Indeed, there is a substantial evidence base that demonstrates WATs are effective in increasing PA in a wide range of populations [16–18], including hospital inpatients and outpatients, with secondary improvements in clinical outcomes such as physical function, pulmonary function, waist circumference, and cardiorespiratory fitness [19–23].

While the evidence supporting WAT use for improving PA is compelling [16–23], most trials focus on efficacy without addressing the practical challenges of implementation and ongoing use in hospital care. Despite widespread interest and enthusiasm for using WATs in healthcare settings, their uptake and continued use are hindered by barriers relating to cost and usability, time constraints and competing demands of clinicians, limited knowledge among both clinicians and patients, and a lack of protocols that are adapted for each healthcare setting [24–26].

Addressing these barriers and evidence-practice gaps to routine WAT use in hospital settings requires approaches guided by implementation science. The study presented in this manuscript evaluates the feasibility of implementing WATs in hospital rehabilitation following an approach that was developed from our prior works on this topic guided by implementation science. This is an essential step in the larger body of evidence focussed on WAT implementation. Earlier work on this topic has sought to understand implementation barriers and identify possible solutions [24, 25, 27–29], and the work presented in this manuscript is the first study of its kind to bring this together and explore the practicalities of implementing WATs in clinical practice. It will extend understanding of the implementation process and evaluate readiness to scale up. The work presented evaluates feasibility in two different rehabilitation services at the hospital, providing a unique contribution to this field of inquiry.

This feasibility trial, guided by implementation science principles, set out to implement and evaluate WAT use in inpatient and outpatient rehabilitation wards at a major teaching hospital. This study used the Fitbit Inspire 3 (Google LLC; San Francisco, CA, US), which provided real-time step count feedback to patients and clinicians. Fitbit devices are a commercially available activity tracking devices which have demonstrated acceptable validity and reliability in stroke, rehabilitation, and older adult populations [30–34], with step count generally being the most reliable metric [35]. 

The aims of this feasibility trial were to:

(1) evaluate the feasibility of implementing WATs in rehabilitation by assessing patient uptake rates, participant retention rates, and device loss rates;

(2) examine patient and clinician perceptions of and satisfaction with WAT use during rehabilitation;

(3) understand the factors that support or hinder WAT use by clinicians in rehabilitation services; and.

(4) assess changes in physical activity levels throughout rehabilitation as measured by WATs.

It is envisaged that the findings from this study will provide deeper insights on the process of implementing WATs in hospital settings, and contribute to the evidence base that explores their implementation on a larger scale.

## Methods

### Study design

A single-arm, pilot feasibility trial was conducted in two rehabilitation services at Flinders Medical Centre (Adelaide, Australia): a virtual rehabilitation ward (VRW) and an outpatient rehabilitation (OR) program. The VRW is an innovative program where patients are admitted as hospital inpatients but receive their care at home. Instead of staying in a hospital ward, patients remain in their own homes while receiving comprehensive inpatient rehabilitation through a combination of telehealth and home visits. Each patient is provided with a technology kit for remote monitoring and telehealth consultations, along with necessary rehabilitation equipment (e.g., exercise equipment, chairs). Admissions typically last 2–3 weeks, and care is delivered by medical specialists, nurses, and various allied health disciplines. As an inpatient rehabilitation service, the VRW provides rehabilitation following acute hospital admissions with the general objective of restoring function sufficient for discharge from hospital. The OR program is a traditional outpatient rehabilitation service where patients attend appointments at the hospital’s rehabilitation department. Admissions typically last 6–8 weeks, with patients receiving care from allied health professionals (i.e. physiotherapy, exercise physiology, occupational therapy). For physiotherapy and exercise physiology, patients complete 2–3 supervised therapy sessions per week in group-based or one-on-one formats. As an outpatient service, the OR program provides rehabilitation for patients who have been discharged from inpatient rehabilitation and are living at home in the community, with the general objective of improving function to support independence and participation in activities of daily living.

This implementation-focussed feasibility study was guided by the Consolidated Framework for Implementation Research (CFIR) [36]. The CFIR offers a framework for identifying factors that influence implementation of evidence-based innovations in healthcare across five domains: intervention characteristics, outer setting, inner setting, stakeholder characteristics, and implementation processes. We conducted a series of works guided by the CFIR [19, 24, 27, 29] to understand requirements for effective WAT use in hospitals, which involved collaborating with clinicians to identify key implementation elements and developing a protocol and supporting resources. The study presented in this manuscript represents the next step by implementing our protocol and resources in the real world, and evaluating if the developed approach can be feasibility and practicably delivered in real-world rehabilitation.

This trial was registered with the Australian New Zealand Clinical Trials Registry (ANZCTR) (Trial ID: ACTRN12624000447550; registration date: 11 April 2024). Ethical approval was provided by the Southern Adelaide Local Health Network Human Research Ethics Committee (HRE00325), and the University of South Australia Human Research Ethics Committee (206100). The WAT device and software used for this project were approved by the Digital Health South Australia department in the Southern Adelaide Local Health Network prior to commencing the study. Written informed consent was provided by all participants. This trial has been conducted and reported in accordance with the CONSORT (Consolidated Standards of Reporting Trials) 2010 guidelines extension to randomized pilot and feasibility trials [37]. A completed CONSORT checklist is available in Supplementary File 1.

### Participants

Participants were patients admitted to the VRW and the OR program. A consecutive sampling strategy was used, with patients meeting the eligibility criteria within the 12-week recruitment period invited in consultation with their treating clinicians. All new admissions to VRW and OR were pre-screened against the eligibility criteria by KS and a senior physiotherapist or exercise physiologist from each service. Clinicians confirmed eligibility during their assessments and gauged interest in participation. KS then approached interested patients with full study information formal invitation.

Sample sizes of 10–15 are recommended as adequate for single group pilot and feasibility studies null [38]. Based on this and consultation with senior staff from the target services who estimated 5–7 new admissions per week, we set a target sample size of *n* = 30 (*n* = 15 per service). We estimated that one-third of new admissions would be eligible and agree to participate based on estimates from other feasibility studies that provided PA interventions in hospital settings [39, 40], and therefore ran the study over a 12-week recruitment period to allow enough time to meet our target sample size.

#### Eligibility criteria

##### Inclusion

Participants were eligible if they were ≥ 18 years old, able to ambulate independently or with stand-by assistance or supervision (as assessed by clinicians), and had a rehabilitation goal related to mobility and physical independence. For VRW participants, the expected length of stay needed to allow for at least 7 days of WAT use, accounting for the time needed for consent processes and device setup early in admission.

##### Exclusion

Participants were ineligible if they had any the following: Lower limb amputation, delirium or cognitive impairment impacting ability to follow instructions or engage with independent behaviour change, not expected to ambulate during admission, insufficient English language to comprehend instructions regarding WAT use or no available interpreter, inadequate vision or dexterity to use devices and no available carer to help, any absolute contraindication to partaking in exercise (e.g. symptomatic severe aortic stenosis, unstable angina).

### Intervention

The intervention protocol and resources were developed via co-design through three workshops with 26 clinicians from Flinders Medical Centre [29]. Prior to implementation, VRW and OR clinicians received training on WAT use, monitoring software, and goal-setting approaches. Support from the researcher and the online resources was available throughout the implementation period, and the online resource is available in Supplementary File 2.

Participants received a Fitbit Inspire 3 (Google LLC; San Francisco, CA, US) to wear on their non-dominant wrist during their rehabilitation admission. The Fitbit Inspire 3 provided step count data, which was used by clinicians to set daily step goals, provide feedback and track progress toward goals throughout patients’ admission. Participants were encouraged to keep track of their daily step counts and try to reach step goals throughout their rehabilitation. This device was selected during a preceding co-design study [29] for its simplicity, support for behaviour change techniques like self-monitoring and goal-setting, water resistance, ease of cleaning, compatibility with existing hospital technology, and relatively low cost compared to other consumer-oriented devices. The associated Fitabase software (Small Steps, Inc; San Diego, CA, USA) enabled clinicians access to multiple patients’ PA data simultaneously and remotely, which was considered important for clinical WAT use. The device was paired with the Fitbit application, accessed via the rehabilitation kit iPad for VRW participants or via personal smartphone/borrowed hospital iPad for OR participants. WATs were provided within the first few days of admission and collected prior to discharge. Assistance was provided with donning and doffing devices by either the researcher or clinicians throughout participation as required (i.e. if dexterity limited ability to independently don/doff).

The lead researcher (KS) set up and provided all participants with their WAT, and provided verbal instructions and demonstration of how to wear the WAT, how to monitor step counts on the device and the associated application, how to sync the device with the application, and charging. Participants were also given an instruction book for the duration of their participation. The physiotherapists and exercise physiologists from each service set step goals with participants in subsequent therapy sessions and encouraged them to track progress on the device and application, and try to meet goals. Goals were gradually progressed or modified throughout the participants’ therapy based on a step goal guide created for the study (available in Supplementary File 2). The step goal guide was informed by findings from the co-design study, which indicated that goals should be tailored to patients’ baseline activity levels, guided by clinical reasoning, and not uniform across all patients [29]. It was also informed by average daily step counts reported in studies using WATs in rehabilitation populations similar to those seen in the VRW and OR services [41–44]. Clinicians could review patients’ activity remotely using Fitabase software (Small Steps, Inc; San Diego, CA, USA). Adherence could also be monitored in Fitabase by viewing wear time, with reminders given to patients who were identified as not wearing their device the instructed amount.

### Outcomes

#### Participant characteristics

Baseline characteristics included age, sex, reason for admission, comorbidities, and use of walking aids at baseline. Length of admission and length of monitoring with WAT were collected at discharge.

#### Feasibility

Feasibility was assessed by evaluating the following outcomes:

- Patient eligibility (% of total patients admitted who were eligible for participation).
- Patient uptake (% of eligible patients who agreed to participate).
- Participant retention (% of participants still wearing WAT at follow-up).
- Device loss (% of devices lost or broken).
- Data completeness (% of participants with mean 12 h of wear time per day).
- Adverse events (incidence and nature of).

#### Patient perceptions and satisfaction

Patient perceptions and satisfaction with using WATs during rehabilitation and the supporting resources were assessed using a brief survey. The survey was developed based on surveys used in other feasibility studies [39] and comprised 10 Likert-scale questions about WAT experiences and four optional open-response questions. Participants completed the survey within 2 days of discharge, with two follow-up reminders at weekly intervals. The 5-minute survey was available electronically (via Qualtrics on rehabilitation iPads for VRW patients or email for OR patients) or in paper format. Patients completed questionnaires independently or with a carer/family member. Where patients required assistance (i.e. due to dexterity) and did not have a carer/family member available, an allied health assistant from the hospital helped the patient with entering responses on the iPad or by hand.

#### Clinician perceptions and satisfaction

Clinician perceptions and satisfaction with protocol implementation, and utility for using WATs in rehabilitation were assessed with a brief survey. The survey comprised five Likert-scale questions about WAT use, each with an optional open-response question. The survey was developed based on surveys used in other feasibility studies assessing clinician experiences of delivering PA interventions [39]. The 5-minute survey was delivered via Qualtrics and distributed via email following the implementation period, with two rounds of reminder emails sent at one-week intervals.

#### Physical activity

Physical activity for participating patients was measured using the Fitbit Inspire 3, which was used as the WAT for the intervention. This WAT device uses a tri-axial accelerometer to measure bodily movement, and provides daily activity data for step counts, minutes of PA at different intensities, minutes of sedentary behaviour, distance moved, floors climbed and calories. It has demonstrated acceptable validity and reliability in stroke, rehabilitation, and older adult populations [30–34]. Fitabase software (Small Steps, Inc; San Diego, CA, USA) was used to manage PA data collected by the Fitbits. Daily step counts, daily minutes of moderate-to-vigorous physical activity (MVPA) and light physical activity (LPA), and daily minutes of sedentary behaviour (SB) were used as outcomes in this study. The primary purpose of collecting PA data was to assess whether WATs could sufficiently capture activity metrics during rehabilitation and support patient engagement in increasing activity levels. These data were not collected to evaluate clinical status, as this study focussed on feasibility of implementation.

All participants wore the WAT on their wrist for the duration of their admission until discharge, which was provided as early as possible after obtaining consent. Participants were instructed to wear the WAT as often as possible throughout the monitoring period, and for a minimum of 12 h each day while awake. PA data was continuously collected while the device was being worn.

### Data analysis

Participant characteristics, feasibility data, and quantitative data from survey were analysed using descriptive statistics. For the patient survey, ‘agree’ and ‘strongly agree’ responses were combined as ‘agree’ for descriptive reporting. Qualitative data from surveys were thematically analysed and organised into emergent categories. For PA data, means and standard deviation (SD) for baseline and follow-up were calculated for each service.

Baseline daily PA data (step counts, MVPA, LPA, and SB) were calculated as the average of the second and third day of WAT wear. Follow up PA data were calculated as the average of the final two days with valid data prior to discharge. The first day and discharge day were excluded from baseline and follow-up averages as they did not represent complete days of WAT wear.

Results from each service were analysed and presented separately due to anticipated differences in walking capacity amongst patients in inpatient vs. outpatient care settings, differences in the way that rehabilitation was provided (combination of telehealth and in-person in patients’ home vs. in-person at hospital gym), and the expected length of admission (approx. 2 weeks vs. approx. 6 weeks). Changes in daily step count, minutes of MVPA, minutes of LPA and minutes of SB from baseline to follow up were tested for normality using the Shapiro-Wilk test and Kolmogorov-Smirnov test. Variables that were normally distributed were compared using a paired t-test, while non-parametric data were compared using the Wilcoxon signed-rank test. Effect sizes were calculated, using Cohen’s *d* for parametric variables (interpreted effect sizes as small (*d* = 0.2), medium (*d* = 0.5), and large (*d* = 0.8) [45] and the matched-pairs rank-biserial correlation (r_W_) effect size for non-parametric variables. This was calculated using the formula r_W_ = Z/√N, where Z is the standardized test statistic), and interpreted as small (r_W_≈0.1), moderate (r_W_≈0.3), or large (r_W_≈0.5) [45]. As recommended for feasibility studies [37], interpretation focused on effect sizes rather than statistical significance. We did not examine potential confounding factors as the study was not designed to test causal effects, the sample size was too small for meaningful adjustment, collecting extra covariates would add unnecessary burden, and any analyses would be statistically unreliable [46, 47]. Missing data were not included in the analyses. Statistical analyses were completed using IBM SPSS 28.0.

## Results

### Feasibility data

#### Recruitment and retention

**Fig. 1:**
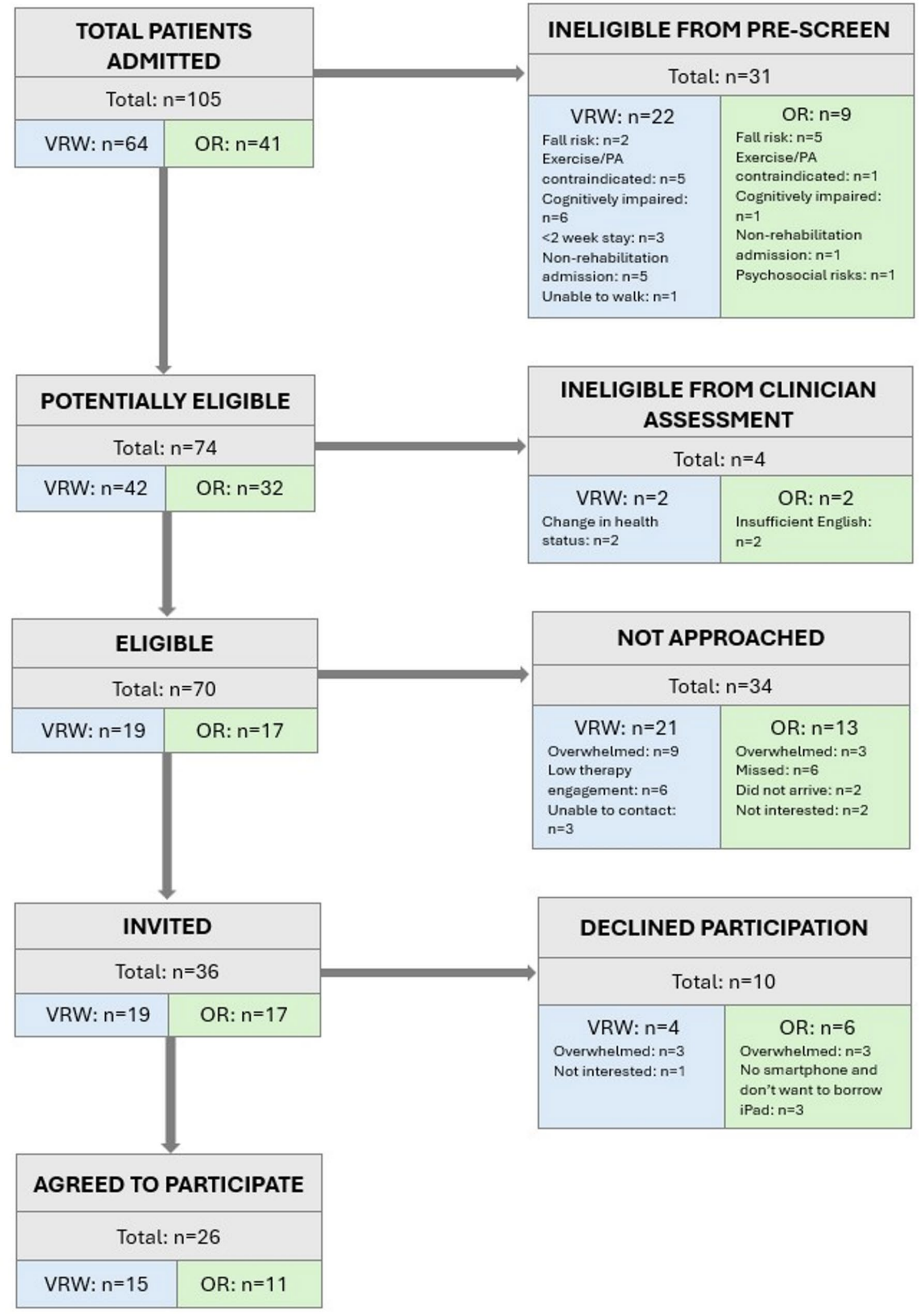
CONSORT flow diagram

Recruitment took place from May to August 2024. One hundred and five patient admissions were screened, of whom 50% were female. Seventy patients were confirmed as eligible during clinician assessment (eligibility rate = 67%), of whom 36 were approached, and *n* = 26 agreed to participate (uptake rate = 72%). The remaining 34 eligible patients were not invited, primarily because they were either overwhelmed by their medical circumstances and rehabilitation demands (VRW: *n* = 9, OR: *n* = 3), or showed low engagement in rehabilitation (VRW: *n* = 6) with clinicians concerned that invitation to the research study could further overwhelm or disengage them. Additionally, scheduling conflicts limited opportunities to invite six eligible patients in the OR program and three patients in the VRW. Ten patients declined participation, citing reasons such as feeling overwhelmed (VRW: *n* = 3, OR: *n* = 3), lack of a smartphone or unwillingness to use a hospital iPad (OR: *n* = 3), and lack of interest (VRW: *n* = 1) (Fig. 1).

All participants in the VRW used the hospital iPads in their rehabilitation kits to access the Fitbit application. In the OR program, 10 participants used their own smartphone to access the Fitbit application, and one participant borrowed a hospital iPad.

All but one participant completed the study, who withdrew due to a medical issue requiring acute hospital admission. They did not rejoin the study upon returning to rehabilitation as this was at the end of the implementation period, but did complete questionnaires assessing satisfaction and provided adequate PA data from their admission.

#### Data completeness

Twenty-four participants (92%) provided complete data (> 12 h average daily wear time). One participant’s WAT was faulty and did not detect wear time despite their attempts to resolve the issue by adjusting the tightness of the wristband. No devices we broken or lost during the study.

#### Adverse events

There were three adverse events during the study, all unrelated to WAT use. This included infection of a prosthesis requiring emergency treatment (*n* = 1), a fall due to dizziness (*n* = 1), and re-admission to acute hospital care for internal bleeding (*n* = 1).

### Participant characteristics

The mean age of participants was 72.6 years (SD: 13.3, range 33–93), and 69% of participants were male. Most participants were admitted to rehabilitation following acute medical admissions (*n* = 11) or falls (*n* = 7). Other reasons for admission to rehabilitation were following acute admissions for fractures (*n* = 2), cancer (*n* = 1), and neurologic conditions (*n* = 1). The average number of comorbidities was 5 (SD: 3). Four participants in the VRW, and *n* = 5 in the OR program mobilised unaided at the start of their admission. The mean length of admission was 17 (SD: 4) days for participants in the VRW, and 46 (SD: 9) days for participants in the OR program. The mean length of monitoring with WATs was 12 (SD: 4) days in the VRW, and 37 (SD: 11) days for participants in the OR program. Full participant characteristics are in Table 1.

**Table 1:** Participant characteristics

		Virtual Rehabilitation Ward (VRW)	Outpatient Rehabilitation (OR)	Combined
n=		15	11	26
Age, median (range)		74 (51–87)	75 (33–93)	74 (33–93)
Age, Mean (SD)		70.9 (11.5)	72.6 (15.9)	72.6 (13.3)
Sex n= (%)				
Male		10 (67)	8 (73)	18 (69)
Female		5 (33)	3 (27)	8 (31)
Reason for admission				
Medical		7	4	11
Falls		1	6	7
Stroke		3	1	4
Fracture		2	0	2
Cancer		1	0	1
Neurologic		1	0	1
Number of co-morbidities				
Mean (SD)		5 (2)	6 (4)	5 (3)
Mobility at start of admission (n=)				
Independent unaided		4	5	9
Single point Stick		2	1	3
4-point stick		1	0	1
2x single point stick		0	1	1
Rollator frame		3	0	3
Four wheel walker		5	3	8
Length of admission (days)				
Mean (SD)		17 (4)	46 (9)	
Length of monitoring period (days)				
Mean (SD)		12 (4)	37 (11)	

### Patient satisfaction and experiences

**Fig. 2:**
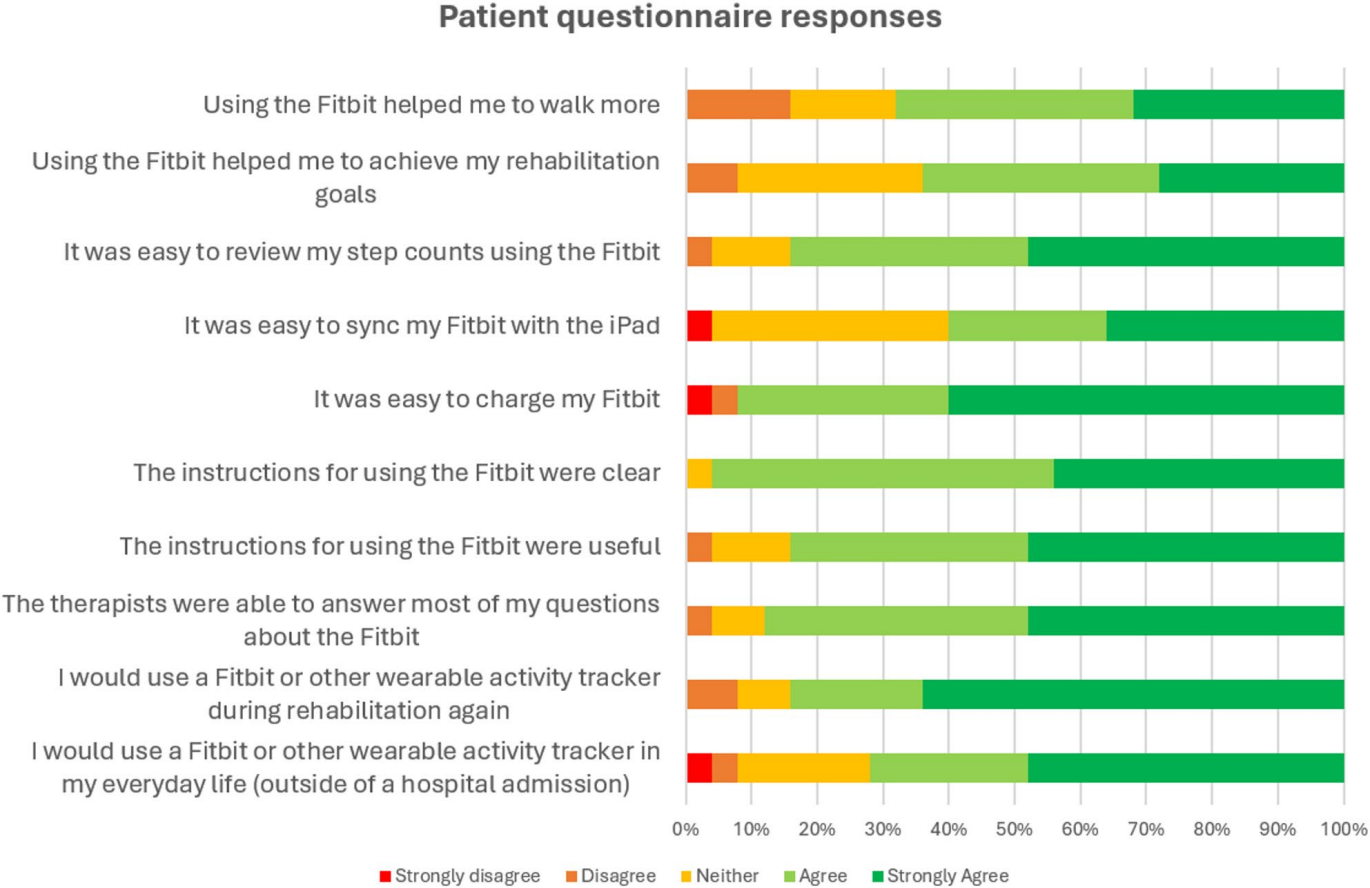
Patient questionnaire responses

Twenty-five (out of 26, 96%) participants completed the end of study survey on their satisfaction using WATs during rehabilitation (*n* = 14 from the VRW, and *n* = 11 from the OR program). Most participants perceived that using the WAT during rehabilitation helped them to walk more (68% agreed or strongly agreed) and helped them to achieve their rehabilitation goals (64%), with a higher proportion of participants from the OR program feeling this way (82% for walking more and 73% for achieving rehabilitation goals) compared to the VRW (57% for walking more and 57% for achieving rehabilitation goals). Most participants (84%) found it easy to review their step counts on the WAT and found it easy to charge the device (92%), with less finding it easy to sync it with the Fitbit application (60%). Almost all participants found the instructions for using the WAT were clear (96%) and useful (84%), and that clinicians were able to answer their questions about using the WAT (88%). Most participants reported that they would use a WAT again (either a Fitbit or other device) during rehabilitation (84%) or in their personal life outside of their hospital admission (72%). Patient questionnaire responses are presented in Fig. 2. The study did not assess specific device preferences, suggesting a general acceptability of WATs.

Sixteen participants provided open responses about their experience using WATs during rehabilitation. Of these, fifteen reported what they most liked about using WATs, with *n* = 5 liking the ability to track their daily step counts, *n* = 5 saying it gave them extra motivation to move more, *n* = 2 liking that the device was easy to use, and *n* = 1 each liking that the device was a watch and waterproof, and that they felt they were making a contribution to research. Five participants reported what they liked the least about using the WAT in rehabilitation, with most of these (*n* = 4) reporting on challenges with dexterity and handling the device (such as charging), and *n* = 1 perceiving that the steps counted were inaccurate compared to their own smartphone’s activity tracking feature. Nine participants provided responses relating to procedures and instructions. Most reported being satisfied and felt that procedures (*n* = 6) and instructions (*n* = 5) did not require changes. Three felt functions of the specific device used could be improved (i.e. watch face staying ‘on’ longer, perceptions of accuracy), and one felt that the application was difficult to navigate.

### Clinician satisfaction and experiences

Of 11 clinicians who were invited to complete the clinician survey, six (VRW: *n* = 3; OR: *n* = 3) responded, which included three physiotherapists and three exercise physiologists. Clinicians who provided responses were aged between 25 and 37, ranged from junior (1–5 years in profession: *n* = 2) to senior roles (10–15 years in profession: *n* = 2), and were an equal split of male and female (*n* = 3 each). Only one clinician had previously used WATs with patients in clinical settings.

**Fig. 3:**
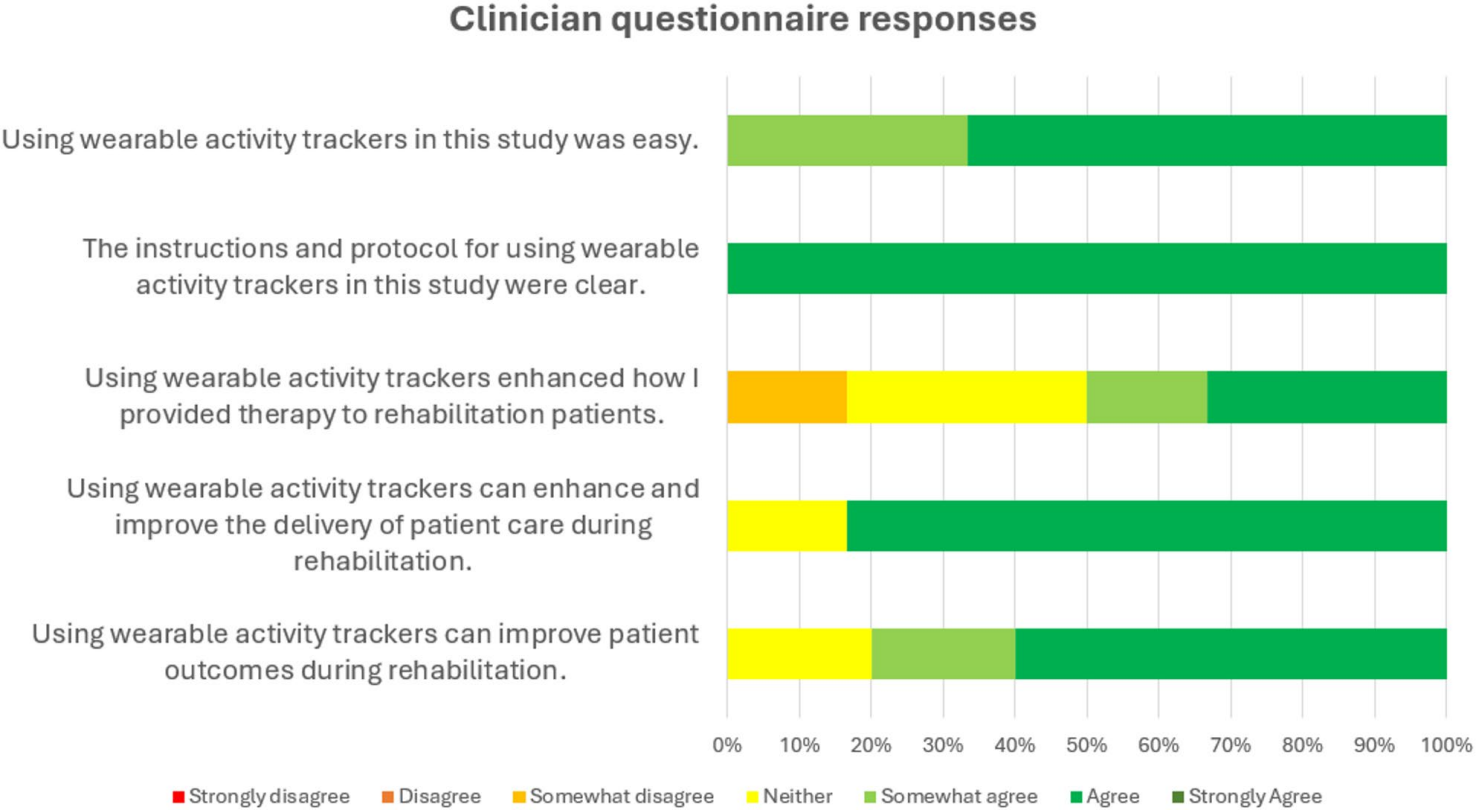
Clinician questionnaire responses

All clinicians (*n* = 6) agreed that using WATs was easy, and that the instructions and protocol for using WATs were clear. Clinicians reported mixed perceptions of WATs enhancing how they provided therapy to patients during the study, with half (*n* = 3) agreeing that WATs enhanced therapy delivery, *n* = 2 neither agreeing nor disagreeing, and *n* = 1 somewhat disagreeing that WATs enhanced their therapy delivery, noting that they would encourage patients to increase their activity regardless of whether WATs were being used and they felt the difference was the availability of objective data. Most (*n* = 5) agreed that using WATs can improve the delivery of patient care and improve patient outcomes during rehabilitation in general, and *n* = 1 was impartial. Clinician questionnaire responses are presented in Fig. 3.

Open responses were provided for each question and provided further context for two overarching categories: (1) challenges of using WATs, and (2) perceptions on the benefits and value of using WATs in rehabilitation. The main challenges of using WATs reported related to using them in a research study (i.e. reviewing eligibility and recruiting participants being inefficient, and creating an extra sense of burden for patients), with other reported challenges related to clinicians having different perspectives on which patients were suitable to participate, and clinicians’ time constraints with reviewing activity data in some instances (i.e. group therapy classes). Perceptions on the value and benefits that WATs could add to rehabilitation related to the provision of objective data for patient PA, their ability to support behaviour change related to PA, enhancing care delivery where face-to-face interactions with patients are limited, and being easy to use. A full summary of themes from open responses is available in Supplementary File 3.

### Physical activity

Complete PA data were available for 24 out of 26 participants, with 14 participants in the VRW and 10 in the OR. Average daily step counts for participants in both rehabilitation services increased from baseline to follow up. The VRW group increased from 1,433 (1158) to 1819 (1398) steps per day, demonstrating a large effect (rW = 0.595). The OR group showed a similar pattern, increasing from 5062 (1548) to 5983 (1622) steps per day, also showing a large effect (rW = 0.532).

Minutes of PA also improved across both services. In the VRW, daily MVPA increased from 0 (1) to 4 (3) minutes, showing a large effect (rW = 0.488), while LPA increased from 95 (53) to 100 (42) minutes, showing a medium effect (rW = 0.344). The FIT group demonstrated similar improvements, with MVPA increasing from 20 (24) to 25 (13) minutes daily, showing a medium effect (rW = 0.435), and LPA increasing from 189 (44) to 218 (59) minutes daily, also showing a medium effect (d = 0.564).

Different patterns for SB were seen between the two services. The VRW group showed a negligible increase in daily minutes of SB from 839 (310) to 853 (250) minutes (d = -0.056). In contrast, the OR group showed a medium-sized decrease in sedentary time from 881 (259) to 770 (241) minutes daily (d = -0.589). Differences in PA between baseline and follow-up measurements for each service are presented in Table 2.

**Table 2:** Physical activity results

		Baseline Mean (SD); range	Follow-up Mean (SD); range	Baseline Median (IQR)	Follow-up Median (IQR)	Effect size: Cohen’s d	Effect size: Matched-pairs rank-biserial correlation (*r*_W_)	*p*-value
VRW *n* = 14	Steps per day	1433 (1158); 272–4117	1819 (1398); 283–5567	1050 (1153)	1366 (1719)	-	0.60 “large”	0.026w
	MVPA min per day	0 (1); 0–4	4 (3); 0–34	0 (0)	0 (4)	-	0.49 “large”	0.068w
	LPA min per day	95 (53); 30–217	100 (42); 21-1289	77 (53)	100 (53)	-	0.34 “medium”	0.198w
	SB min per day	839 (310); 408–1326	853 (250); 399–1289	849 (568)	814 (261)	-0.06 “negligible”	-	0.839t
OR *n* = 10	Steps per day	5062 (1548); 2614–8120	5983 (1622); 3497–8757	4749 (1159)	5986 (1590)	-	0.53 “large”	0.093w
	MVPA min per day	20 (24); 0–71	25 (13); 8–47	12 (16)	23 (19)	-	0.44 “medium”	0.169w
	LPA min per day	189 (44); 122–250	218 (59); 134–339	175 (59)	212 (58)	0.56 “medium”	-	0.108t
	SB min per day	881 (259); 434–1242	770 (241); 328–1079	855 (324)	866 (310)	-0.59 “medium”	-	0.095t

Legend: VRW = virtual rehabilitation ward; OR = outpatient rehabilitation; SB = sedentary behaviour; LPA = light physical activity; MVPA = moderate to vigorous physical activity; SD = standard deviation; IQR = interquartile range. Superscript t = p-value derived from paried t-test; superscript w = p-value derived from Wilcoxon signed rank test

## Discussion

This feasibility study demonstrated that WATs can be successfully implemented into hospital rehabilitation services. Overall, implementation was feasible, with high uptake rate among invited patients, excellent retention, high data completeness, no device loss, and no adverse events related to WAT use. Patient satisfaction was high, with most participants perceiving that WATs as helpful, and the majority expressing willingness to use WATs again in rehabilitation. While clinicians unanimously found WATs easy to use and most believed they could improve care delivery, they described implementation challenges including time constraints and varying perspectives on patient suitability. In both services, PA data showed encouraging trends, with large effect sizes for increased daily steps in both the VRW (1433 to 1819 steps/day, r_W_=0.595) and OR service (5062 to 5983 steps/day, r_W_=0.532).

This study demonstrates a feasible approach for implementing WATs as a regular part of rehabilitation, and potential for scale up. Numerous factors contributed to the success of this feasibility study. These included using a basic, user-friendly WAT, having a simple protocol, instructions presented in a combination of physical and online resources with demonstrations provided, using remote monitoring, and using a simple and easy to understand metric (daily step counts) to track and promote PA. These approaches were intentionally selected based on previous works that were guided by implementation science [27, 29]. Our work focused on understanding both the innovation (using WATs in clinical settings) and its implementation context (including the setting, processes, and different users involved). This enabled identification of essential elements while allowing adaptation to setting-specific needs and constraints, which is critical for successful implementation of healthcare innovations [48, 49]. Thus, this study supports both previously identified elements and the developed protocol, while demonstrating the value of a comprehensive, collaborative approach to implementation.

While this study demonstrated feasibility in the approach to using WATs, recruitment presented interesting insights for future implementation. Uptake was high when patients were invited, reflecting strong interest. However, clinical teams made considered decisions about approaching patients, prioritising patient wellbeing and engagement with standard rehabilitation. As a result, many patients meeting the eligibility criteria weren’t approached. Participating patients generally reported positive experiences, though it is possible that selection bias influenced this result [50]. These recruitment challenges suggest that recruitment models relying on individual patient invitation, common in trials with similar populations and interventions [39, 40, 51, 52], may inadvertently introduce a perceived burden disproportionate to the actual burden of participation. Alternative recruitment methods, such as centre-based enrolment or other systematic approaches, may enable inclusion of broader samples while maintaining appropriate clinical oversight.

Patient satisfaction and engagement was high in this study. Most participants were > 65 years, and these findings challenge common assumptions about older adults’ ability or willingness to engage with technology. Consistent with our results, previous studies that use WATs with community-dwelling older adults have demonstrated their utility, acceptability, ease of use, and positive impact on PA awareness and motivation [53], highlighting the viability of using WATs with older adults. Simple and user friendly devices and clear instructions have been identified as key success factors for older adults using WATs in community settings [53, 54], and our findings suggest these factors are equally important in hospital settings.

Clinicians found using WATs to monitor patient activity and set daily step goals easy, felt WATs could benefit rehabilitation, and noted that having objective data to inform goal setting and feedback supported interactions with patients focussed on promoting activity. Perspectives varied on how WATs enhanced therapy delivery, which may have been influenced by constraints of services and workflows. Challenges described by clinicians related to inefficiencies of the recruitment approach, and having limited opportunities and time to review step counts and goals in group-based sessions. This aligns with recognised challenges in healthcare technology adoption, particularly regarding workflow integration, where clinicians working in time constrained conditions with numerous competing priorities may have limited capacity for adopting new approaches to care delivery [55]. Previous research has identified this as a key consideration in implementing WATs in clinical settings [24], which may explain the diverse perspectives on therapy enhancement despite consistent positive experiences with WAT usability and potential clinical value. Ensuring future efforts to implement WATs are efficient and cohesive with clinician workflow and providing clinicians with adequate training and support will be essential to successful workflow integration.

Daily step counts, daily minutes of LPA, and daily minutes of MVPA increased from baseline to follow-up for patients in each service. Wearable activity trackers contain several effective behaviour change strategies [56], such as goal-setting, feedback, and self-monitoring. These behaviour change techniques were central to how WATs were used in this study, and likely influenced patients’ mobility behaviours. The observed increases in PA are consistent with the wider evidence base for PA promotion using WATs, which consistently demonstrates their effectiveness in hospitalised, outpatient, and other clinical and healthy populations. While this study adds to the broader evidence base for using WATs for PA promotion in clinical settings, our findings for changes in PA should be interpreted cautiously, given the feasibility nature of this trial [16–19, 57]. Increased PA levels of 415 steps per day in the four weeks following a hospitalisation [58], and 15 min of LPA during rehabilitation [59] are typical, and this study did not have a comparison group to evaluate the specific effect of WATs on PA. Future work that builds on this feasibility trial should undertake evaluations of PA outcomes in powered samples and against comparator groups to ensure changes are akin to those of the wider evidence base for WATs in healthcare [16, 19–21, 23, 44].

### Strengths and limitations

Unlike prior studies that focus on WAT effectiveness, this study addresses the evidence-practice gap by testing how WATs can be practically embedded in hospital workflows. A key strength of this study was its implementation across two contrasting rehabilitation services, enabling evaluation of the same approach in settings with distinct operational constraints. The services differed notably in patients’ rehabilitation stages, mobility status, the amount and type of contact with clinicians, admission length, and technology provision (hospital provided iPads in VRW versus patients’ personal devices in OR). Despite these differences, feasibility was demonstrated in both settings, suggesting that the approach used in this work may be suitable for a variety of settings without extensive adaptations. Another important strength was the study’s theoretically informed development process. Development of this study was guided by implementation science and the CFIR [36], which involved understanding the innovation, the context in which it would be applied, and the process of implementation. We identified critical elements of using WATs in healthcare, and used a co-designed approach to adapt this for the target setting [27–29]. This comprehensive, evidence-based approach to implementation appears to have contributed substantially to the success of this study.

There are also some limitations to this study. Firstly, half of eligible patients admitted were not approached, many due to assumptions about their willingness or ability to participate due to being overwhelmed by their condition or disengaged with therapy. These patients may have had different experiences and outcomes from using WATs, and the findings from this study should be considered with this in mind. Similarly, only half of the clinicians invited completed the clinician survey, which may have introduced self-selection bias in the results [50]. It is possible that other clinicians involved had different experiences than those who provided responses, and the responses from the clinician survey should therefore be considered carefully. We did not conduct qualitative interviews with patients or clinicians as part of this study, which would have provided more in-depth detail on patients’ and clinicians’ experience and perceptions of using WATs during rehabilitation. This study also had a 3-month recruitment period, and it is possible the findings may not be generalisable to longer or sustained implementation periods. Given evidence that commercial WATs can be imprecise or misclassify step counts [60], our step-count findings should be interpreted with caution. Finally, as a pilot feasibility study, this study was not powered to evaluate the effectiveness of this approach to using WATs for increasing PA. Participants were also not blinded which may have introduced performance bias and contributed to overestimation of satisfaction with WATs, and it lacked a control group to make comparisons of observed changes in PA.

### Future directions

Future iterations of this work may benefit by adopting service-level or organisation-wide implementation approaches, with all patients being recruited upon admission with the ability to opt-out, rather than individual opt-in patient recruitment. Such an approach in implementation trials may better reflect real-world use, overcome the recruitment challenges identified in this study, and allow for a wider range of experiences to be captured. The results of this study also highlight the potential for integrating WATs in inpatient and outpatient rehabilitation on a larger scale. While Fitbit devices (selected during co-design for their ease of use, comfort, and patient feedback features) and Fitabase monitoring software (providing a convenient clinician dashboard) worked well in this feasibility study, the monthly per-participant cost of Fitabase could present a barrier to large-scale implementation. Use of lower-cost platforms with similar functionality, development of purpose-built WATs and companion software designed specifically for clinical settings, and exploring negotiated costs with developers for licensing at scale could enhance the feasibility of larger-scale implementation.

Future research in this area should evaluate the longer-term implementation of WATs in rehabilitation in larger scale trials with a comparison group, sufficiently powered sample sizes, and collect in-depth qualitative data from patients and clinicians. Buy-in and engagement with WAT use among stakeholders may change over time, and exploring this in detail will be important in understanding what is needed to support sustained use and engagement. Evaluating effects of this approach on PA and other rehabilitation-specific outcomes like physical function will help determine if similar results to the wider evidence base for WATs can be reproduced in real-world applications. Building on this feasibility work, future studies should also evaluate the clinical impact of WAT integration on patient outcomes, such as physical function and mobility, with longer follow-up periods to fully capture potential benefits. Additionally, evaluating this approach in other clinical settings where patient mobility is important, such as acute inpatient hospital wards, will be valuable, noting that adaptations for each setting will be required along with pilot testing and validation of stakeholder questionnaires to ensure suitability for larger scale projects. Subsequent implementation research that builds on this feasibility study should also involve patient representatives in planning and development to ensure that approaches are appropriate and relevant for patients. Finally, evaluating the cost-effectiveness of integrating WATs into different healthcare services will be crucial for healthcare providers and policymakers. While WAT integration requires initial investment in devices and monitoring systems, and some additional clinician time during admission, the potential benefits of enhanced recovery and improved patient outcomes could offset these costs or even lead to downstream cost savings (e.g. through shorter lengths of stay, reduced readmission rates, and better functional outcomes). As such, robust evidence of cost-effectiveness, including both implementation costs and potential healthcare benefits, will be essential to support the broader integration of WATs in standard practice.

## Conclusion

This is the first study that has focussed on real-world WAT implementation, and it demonstrates the feasibility of implementing WATs in inpatient and outpatient rehabilitation to promote patient PA. Recruitment and retention rates were high, and both patients and clinicians reported satisfaction with the approach used and resources provided to support WAT use. Physical activity data completeness was high, and increases in daily step counts, daily LPA and daily MVPA from baseline to follow-up were observed in each service. These findings are an important addition to the evidence-base for clinical WAT use toward implementation, contribute to the growing body of implementation science research on digital health in rehabilitation, and provide a model for integrating WATs into clinical workflows. Factors contributing to success were the simplicity of the WAT, protocol, and supporting resources, all of which were intentionally selected and developed as part of a comprehensive and collaborative body of work informed by implementation science. To realise the potential of WATs in healthcare, future research should move toward scalable implementation. Building on the insights from this study by evaluating implementation at the service level, assessing cost-effectiveness, and developing clinician training strategies to enhance adoption will be essential for ensuring success. Additionally, studies that use service-level implementation strategies to capture a broader range of patient experiences can confirm real-world effectiveness through larger scale trials. Finally, exploring the application of this approach across diverse clinical populations and settings, and examining potential downstream effects of using WATs on rehabilitation outcomes for patients will provide valuable insights on broader WAT implementation in the healthcare sector.

## Supplementary Information

Below is the link to the electronic supplementary material.

Supplementary Material 1

Supplementary Material 2

Supplementary Material 3

## Data Availability

Datasets generated and analyzed during this study are available from the corresponding author upon reasonable request.

## Abbreviations

ANZCTR: Australian New Zealand Clinical Trials Registry
CFIR: Consolidated Framework for Implementation Research
CONSORT: Consolidated Standards of Reporting Trials
LPA: Light physical activity
MVPA: Moderate-to-vigorous physical activity
OR: Outpatient rehabilitation
PA: Physical activity
SB: Sedentary behaviour
SD: Standard deviation
VRW: Virtual rehabilitation ward
WAT: Wearable activity tracker
