# Supplementary material for: Implementation of wearable activity trackers in hospital rehabilitation: a feasibility study tailored to local settings
Source: BMC Health Serv Res. 2026 Jan 24;26:268. doi: 10.1186/s12913-026-14066-4 (PMC12910737; doi:10.1186/s12913-026-14066-4)
Supplement: Supplementary file 2 — Supplementary Material 2 [file 12913_2026_14066_MOESM2_ESM.pdf]

**Aspects of using WATs that were challenging**	**Perceptions on the benefits and value that WATs added to rehabilitation**
**Research processes**<br>- The main challenge was in onboarding participants and setting up devices as this was time consuming and involved several steps/visits on top of standard/routine contact points between clinician and patients. (recruitment)<br>- Having to maintain consistency with wear site for the research processes (same wear site for all patients)<br>- Different perspectives on patient suitability, and what sort of activity was being promoted between some disciplines/clinicians involved (recruitment)<br>- Time taken and many steps involved to recruit and get patients set up. (recruitment)<br>- Cognitive load/burden explaining research processes to patients and obtaining consent, making them disengage. (recruitment)<br><br>**Time constraints**<br>- Useful for brief interactions that it relates to, but challenging to integrate in group settings and where time is limited. (group setting)<br>- Limited time for clinician to review activitiy (reviewing activity)<br>- Limited time and opportunity to discuss in group settings. (group setting)<br><br>**Technical issues**<br>- Occasional tech issues<br><br>**Patient suitability**<br>- For the 'right patient'<br>- Different perspectives on patient suitability, and what sort of activity was being promoted between some disciplines/clinicians involved<br>- Patients who participated were already more engaged in therapy and less sick - more likely to have a good outcome anyway	**Objective PA data**<br>- Objective data supporting provision of education and encouragement to be active.<br>- Provides a metric/information to discuss<br>- Provides patients something specific to aim for. Boost motivation<br>- Objective data for something they are interested in and promoting for therapy (steps/walking).<br><br>**Addition for behaviour change approaches**<br>- Provides patients something specific to aim for. Boost motivation<br>- Assist goal setting<br>- Supported provision of motivating/encouraging patients<br><br>**Enable support for delivering care when less face to face opportunities**<br>- Added benefit of monitoring activity for the VRW where there are less opportunities for seeing patients.<br><br>**Easy to use**<br>- Easy to track steps and access the data.
